# Short- and Long-Term Outcomes of an Adventure Therapy Programme on Borderline Personality Disorder: A Pragmatic Controlled Clinical Trial

**DOI:** 10.3390/brainsci14030236

**Published:** 2024-02-29

**Authors:** Alba Gabarda-Blasco, Aina Elias, Mariona Mendo-Cullell, Laura Arenas-Pijoan, Carles Forné, David Fernandez-Oñate, Laura Bossa, Aurora Torrent, Xavier Gallart-Palau, Iolanda Batalla

**Affiliations:** 1 Psychiatry Service, University Hospital Santa Maria, Avinguda Rovira Roure 44, 25198 Lleida, Spain; agabarda@gss.cat (A.G.-B.); aelias@gss.cat (A.E.); m.mendo91@gmail.com (M.M.-C.); larenas@gss.cat (L.A.-P.); dfernandez@gss.cat (D.F.-O.); lbosa@gss.cat (L.B.); atorrent@gss.cat (A.T.); 2 Heorfy Consulting, 25007 Lleida, Spain; 3 Department of Basic Medical Sciences, University of Lleida, 25006 Lleida, Spain; 4 Biomedical Research Institute of Lleida Dr. Pifarré Foundation (IRB Lleida), 25198 Lleida, Spain; xgallart@irblleida.cat; 5 Neuroscience Area, +Pec Proteomics Research Group (+PPRG), University Hospital Arnau de Vilanova (HUAV), 25198 Lleida, Spain; 6 Psychology Department, University of Lleida (UdL), 25006 Lleida, Spain; 7 Faculty of Health Sciences, Valencian International University, 46002 Valencia, Spain; 8 Medicine Department, Faculty of Medicine, University of Lleida (UdL), 25006 Lleida, Spain

**Keywords:** borderline personality disorder, Adventure Therapy, treatment, clinical trial, longitudinal study

## Abstract

Adventure Therapy (AT) is a therapeutic intervention utilizing the natural environment and adventure activities as tools for psychotherapeutic interventions. It has been demonstrated to be appropriate for the intervention of patients with borderline personality disorder (BPD). This study aims to evaluate the response to AT treatment compared with the response to treatment as usual (TAU), based on cognitive behavioural therapy, in the short and long term, assessing clinical, psychosocial, and functional outcomes; quality of life; and physical health levels. This study extends the sample of and is a follow-up to a pilot study published in 2021, with a sample of 30 patients in the AT group and 10 in the control group. It does not allow us to affirm that AT provides better outcomes than TAU, as the positive effects observed immediately after therapy seem to be attenuated in the long term. Therefore, the effectiveness of long-term psychotherapy did not show differences between AT and TAU therapies in the treatment of BPD patients. However, the effects of intangibles observed during therapy by professionals and patients were not reflected in the measurements collected. Therefore, we believe it is necessary to increase the programme duration, complement treatment with a specific physical health programme, assess results with more specific instruments, and/or move towards a qualitative methodology to measure perceived changes in clinical improvement. New studies are needed to evaluate the results of the proposed changes.

## 1. Introduction

Borderline personality disorder (BPD) affects 0.7% to 2.0% of the population [1], making it the most frequent personality disorder [1]. With a prevalence that may be as high as 20%, it is also the most prevalent personality disorder in clinical settings [2]. BPD is characterized by a dominant pattern of instability in interpersonal relationships, self-image and affect, as well as intense impulsivity. These symptoms typically begin in early adulthood and are present in a variety of contexts. These maladaptive traits cause significant adjustment difficulties that affect various areas of a person’s life, leading to high levels of anxiety, emotional discomfort, and low frustration tolerance [3]. They are sufficiently inflexible and maladaptive, causing significant functional impairment [4]. As a result, individuals with BPD often develop dysfunctional patterns and escape behaviours characterized by immediacy. Maintained over time, these behaviours may result in chronic helplessness, behavioural rigidity, and unstable interpersonal relationships [3], which can increase the risk of suicide, particularly if they have comorbid disorders such as depression or substance use. The rates of comorbidity in people with BPD are significantly higher than in those with other conditions [2]. Depression is the most prevalent comorbid disorder (61%), followed by post-traumatic stress disorder (36%), bipolar disorder (20%), and eating disorders (17%) [1]. Patients may also have physical disabilities due to self-injurious behaviours or unsuccessful suicide attempts [3]. Recent studies suggest that individuals diagnosed with BPD may experience poorer physical health [5,6], potentially due to factors such as obesity, which may increase the risk of medical illness [7]. Additionally, BPD may be associated with poorer psychosocial functioning, including deterioration in work, social relationships, and leisure activities [2], as well as an increased risk of psychiatric morbidity [2] and reduced quality of life [8].

Treating patients with BPD can be challenging [1]. However, research suggests that psychotherapeutic interventions can be effective for individuals with BPD [9,10,11,12]. A key goal of treatment is to help patients identify and modify dysfunctional traits, replacing them with healthier behavioural, emotional, and relational patterns [9].

Adventure Therapy (AT) refers to intervention programmes that use adventure-based activities for therapeutic purposes [13]. This psychotherapeutic intervention method is mainly developed outdoors, with challenges and adventure activities, and implemented by mental health professionals [14]. The most commonly used definition for AT is as follows: “the prescriptive use of adventure experiences provided by mental health professionals, often conducted in outdoor settings, that kinesthetically engage clients at cognitive, affective and behavioural levels” [15]. This method is used in work with adolescents and adults in particular, and it is adapted to the needs of individuals and groups with the aim of achieving social and psychological change [16].

AT is based on the Outward Bound model, developed in the UK by Kurt Hahn, and is part of the experiential learning paradigm [17]. It consists of expeditions in outdoor settings with the aim of challenging participants to overcome their perceived limitations and develop a better sense of self. In addition, challenges are used as a therapeutic tool to help develop new strategies to communicate effectively with each other and to improve their social skills [15,16]. AT activities consist of a specific set of problem-solving tasks that are designed to be challenging and create a state of adaptive dissonance that takes patients out of their comfort zone [18]. Patients usually engage in these unfamiliar tasks in a peer group. As they overcome the challenge by solving the problem, patients experience control and understand the causes and consequences of their actions [17,19]. Eventually, this leads to the development of adaptive skills that emerge as a result of these experiences, which they can incorporate into their daily lives [15].

Seven areas are described that form the basis of AT: action-focused therapy, unfamiliar environment, climate of change, assessment skills, developing small groups with a supportive aspect, focusing on strengths rather than deficits, and the changing role of the therapist [15]. Similarly, there are key elements that differentiate Adventure Therapy from other integrated therapeutic approaches or link it to them: positive influence of the outdoor environment in the therapeutic process, use of eustress (positive response to stress), active and directive use of the patient’s participation and responsibility in their therapeutic process, patient participation and responsibility, participation in meaningful adventure experiences, focus on positive changes in present and future functional behaviour, and an ethic of care and support that is tailored throughout the therapeutic experience [15].

The professionals who carry out this therapy do so from a clinical perspective, combining mental health knowledge and outdoor adventure skills. Therefore, objectives are set that aim to bring about changes in different areas of the patient’s life through the ABC-R model (affect, behaviour, cognition, and relationship) [15].

AT can be applied in different contexts (hospitals, special intervention centres, camps, etc.) and can be implemented through different approaches (cognitive, behavioural, etc.) [15].

The effectiveness of this method has been scientifically proven since the 1960s, particularly in Anglo-Saxon and Scandinavian countries and in Germany [14]. Various national and supranational organizations are developing powerful lines of research, including the American Outdoor Behavioral Healthcare Center, the Australian section of Bush Adventure Therapy Research and Evaluation, and the Scandinavian Nordic Outdoor Therapy Research [14,15]. In Spain, despite its growing importance over the last 15 years, AT remains a rather unknown and innovative approach [20,21]. Not until 2015 was the first AT programme launched in Spain [21].

Several studies support the effectiveness of AT. People with autism spectrum disorders [16,22]; with psychotic spectrum disorders [23,24]; and children, adolescents, and young adults with cancer [25] have benefited from AT. Recent meta-analyses [13,18,26] and reviews [27] present studies on AT in different populations (people at risk or undergoing treatment for behavioural or mental health problems, adolescents, juvenile offenders, people with chronic diseases, substance users, etc.). The results of these studies indicate that the programmes are effective in improving psychological, behavioural, emotional, interpersonal, clinical, and self-concept aspects; that they have a positive effect on the general functioning and psychological well-being of the participants; and that results can be maintained in the long term. Studies suggest caution in generalizing results to all programmes and populations. Further research is necessary to better understand the variability of AT programme outcomes [13,27].

Thus, given the personality traits associated with people with BPD, AT may be appropriate to intervene in aspects with which people diagnosed with BPD experience difficulties [12,26]. AT confronts the person diagnosed with BPD with their difficulties in an experiential manner, which leads to an increase in their discomfort and the emergence of dysfunctional patterns and escape behaviours (experiential avoidance as dysfunctional automatism) [28,29]. This therapy provides them with the opportunity to confront situations that they would typically avoid, thereby enhancing their tolerance to discomfort in situations of frustration and insecurity. It also helps to improve emotional regulation through the use of intrapersonal or interpersonal coping strategies, such as asking for help or expressing discomfort assertively, in real-life situations with the guidance of therapists. The ultimate goal is to integrate the acquired knowledge into the patients’ daily lives [15].

On this theoretical basis, a pilot study of AT for BPD patients called “Afrontant el Buit” (Facing the Void) was launched and showed promising short-term results (healthier lifestyle habits, greater functionality, and better quality of life) [12]. This article [12] describes a pragmatic, controlled, non-randomized clinical trial. The study included patients with a diagnosis of BPD in an Adventure Therapy group and a cognitive behavioural therapy control group. Each group consisted of 10 individuals, matched for sex and age, chosen from the Specific Partial Hospitalization Programme (PHSP) of the Adult Psychiatric Day Hospital of the University Hospital Santa Maria of Lleida [6]. Fourteen weekly sessions were conducted in both groups. The study compared the differences in variables between the AT group and the control group before and after the therapy. The results showed that the AT group had medium to large effect sizes in improving some metabolic variables. In terms of psychometric variables, almost all variables showed better results in the AT group, but the effects were not significant. We are not aware of any other published articles on the effects of AT in patients with BPD.

The objective of the present study was to compare the response to TA treatment with treatment as usual (TAU) based on cognitive behavioural therapy. The study evaluated clinical, psychosocial, and functional outcomes; quality of life; and physical health levels at two time points: pre- and post-treatment assessments in both the TA and TAU groups and assessments 2–3 years after the end of treatment. Based on the reviewed studies and considering the promising short-term results obtained in the pilot study [12], this study proposes two hypotheses: (1) completion of the AT intervention will result in improvement in the mentioned areas compared to the group performing the CBT intervention, and (2) patients who undergo the AT intervention will achieve better results than those who undergo the CBT intervention in the long term (2–3 years).

## 2. Materials and Methods

### 2.1. Design and Population

This was a long-term extension study of a pragmatic pilot clinical trial that evaluated 20 patients with BPD: 10 in an AT group and 10 in a conventional therapy group, based on cognitive behavioural therapy. The study design, population, and results were published in Mendo et al. [12]. We believe that extending follow-up times is important, as some reviews on personality disorders use longitudinal data and conclude that these disorders are moderately stable but also show marked improvement over time. Psychosocial therapies can modify the clinical course of these disorders, leading to improvement [30]. Additionally, there are several longitudinal studies that have observed improvement over time [31,32,33]. Therefore, it is essential to extend evaluations to measure the changes more accurately.

Briefly, the study included patients from the Partial Hospitalization Specific Programme (PHSP) of the Adult Psychiatric Day Hospital at the University Hospital Santa Maria in Lleida. The sample selection for this programme enabled us to include patients who had experienced some symptomatic stabilization while also facilitating a more intensive and multidisciplinary follow-up of patients.

The present study added 20 patients to the AT group and extended the follow-up to 3 years after therapy. Thus, in this study, the AT group consisted of 30 patients, and the control group consisted of 10 patients. The exclusion criteria for the study were limited to participants with an insufficient physical capacity to engage in AT and those who experienced significant clinical deterioration during therapy. Four patients in the AT group withdrew from the study due to clinical worsening.

It is important to note that the reduced number of patients in the study is due to the need for bonding and the psychopathological stability required to carry out the therapy, as well as the limited number of participants admitted to the sessions. Additionally, monitoring during the pandemic period was not possible, and longitudinal follow-up was difficult due to the characteristics of the patients. Therefore, the sample size is valued both in the short and long term.

This study was conducted in accordance with the Declaration of Helsinki and approved by the Drug Research Ethics Committee of the University Hospital Arnau de Vilanova. All participants were fully informed about the study and signed an informed consent form.

### 2.2. Procedure

A total of 14 weekly sessions were carried out with all groups. The number of sessions was determined based on previous studies with BPD patients [34] and the characteristics of the AT sessions [15]. The sessions with the control group consisted of group psychotherapy based on the usual PSHP (Partial Specific Hospitalization Programme) cognitive behavioural therapy [35] for people diagnosed with BPD. These were closed groups with 90 min sessions at the Adult Psychiatric Day Hospital. Patients in the AT group participated in group therapy sessions based on the AT model [15,36]. They were also closed groups with 120 min sessions conducted outdoors with different modalities of physical activity (problem-solving games, slacklining, bouldering, rock climbing, hiking, via ferrata, and kayaking). AT sessions are characterized by experiential learning through the resolution of individual and group challenges, using metaphors in physical activities carried out in an outdoor environment. This learning is then applied to everyday life [15].

Details about the study procedure and the characteristics and objectives of the sessions have been published in Mendo et al. [12]. Generally, sessions begin by establishing a safe environment for patients, followed by introducing a challenge that allows for individual and group choice. The therapist–facilitators offer support based on the coping styles of each patient. Next, a reflection phase ensures the perception of learning, and the session concludes with a transfer phase that focuses on introspection and the practical application of the learning [15].

### 2.3. Measurements

Five assessments were performed. As in the study by Mendo et al. [12], one assessment took place in the two weeks before the first session (pre-treatment) and another in the two weeks after the last session (post-treatment). For the present study, three additional assessments were carried out at 6 months, 12 months, and 2–3 years post-treatment. The initial design spanned 18 months, but these data could not be collected due to restrictions related to the COVID-19 pandemic [37]. Data were collected from medical records, a physical examination, and a semi-structured clinical interview. 

The following clinical and sociodemographic variables were recorded for each patient: gender, age, height, weight, body mass index (BMI), waist circumference, systolic and diastolic blood pressure, drug treatment, frequency of physical activity, tobacco and other drug use, primary and secondary diagnosis according to DSM-5 [3], marital status, cohabitation situation, level of education, occupation, and employment situation. These sociodemographic variables were selected because they are associated with patients who exhibit dysfunctional traits that cause significant difficulties in adaptation and affect various areas of their lives [3]. These difficulties include poorer psychosocial functioning, such as deterioration in work, social relationships, and leisure activities [2]. By assessing the degree of impairment in these areas, we can better understand the patient’s condition.

Recent studies suggest an association between individuals diagnosed with BPD and poorer physical health [5,6]. Therefore, it is relevant to measure variables such as BMI, waist circumference, systolic and diastolic blood pressure, and frequency of physical activity. Furthermore, obesity may link BPD to an increased risk of medical illness [7] and greater psychiatric morbidity [2]. As a result, we have chosen to measure several biological parameters to assess these areas. As a result, the physical activities performed in the AT sessions will also affect these variables. The parameters that we measured are as follows: glucose, total cholesterol, high-density lipoprotein cholesterol (HDL cholesterol), low-density lipoprotein cholesterol (LDL cholesterol), triglycerides, and glycated haemoglobin (HbA1c).

Psychometric variables were also collected. Questionnaires were selected to evaluate various aspects of the clinical presentation of patients diagnosed with BPD [3]. The following self-administered questionnaires were distributed, each of them in their validated Spanish versions. Below is a description of their usefulness and cut-off points:

- Beck’s Hopelessness Scale [38]. This scale subjectively assesses people’s negative expectations about their future. It is also a potential surrogate predictor of suicidal behaviour. It consists of 20 true/false items. The total score varies between 0 and 20 and is the sum of all the items. The cut-off point is set at scores equal to or greater than 9. Scores above these values are considered to be good predictors of suicide risk [39].
- Rosenberg self-esteem scale [40]. This is one of the most widely used scales for the global measurement of self-esteem. It consists of 10 items focusing on feelings of respect and self-acceptance. It is a Likert-type scale ranging from 0 to 3, where the person reflects their level of agreement or disagreement with the items. The total score is the sum of all items and ranges from 0 to 30. Low self-esteem is defined as a score below 15, while higher scores indicate higher self-esteem [41].
- State-Trait Anxiety Inventory (STAI) [42]. This questionnaire consists of 40 items: 20 items to assess state anxiety (A-S) and 20 to assess trait anxiety (A-F). It has a Likert scale from 0 to 3 according to its intensity (state) and frequency of occurrence (trait). Two total scores are obtained, one for A-S and one for A-F, ranging from 0 to 60, with higher scores corresponding to greater anxiety. These direct scores are transformed into quantiles according to gender and age [43].
- Plutchik’s Impulsivity Scale [44]. This scale assesses, among other things, one’s previous suicide attempts and the intensity of their current suicidal ideation. It consists of 15 items scored from 0 to 3 depending on the occurrence of impulsive behaviour. The total score is the sum of all the scores and ranges from 0 to 45. In the Spanish version, the cut-off point is 20; values below that score indicate low impulsivity. The higher the score, the greater the tendency for impulsive behaviour [45].
- World Health Organization Disability Assessment Schedule (WHODAS 2.0) [46]. The 36-item version measuring health and disability was used, which assessed the level of functioning in 6 life domains in the 30 days prior to the test. These domains are cognition, mobility, personal care, relationships, activities of daily living, and participation in society. Each item is rated on a Likert scale from 1 (“no difficulty”) to 5 (“extreme difficulty”). The total score for each dimension is calculated by weighting the questions and the severity levels obtained; the score ranges from 0 (without disability) to 100 (total disability) [47,48].
- Abbreviated World Health Organization Quality of Life Questionnaire (WHOQOL-BREF) [49]. This is an abbreviated version of a WHO questionnaire consisting of 26 questions about quality of life as perceived by patients during the previous 2 weeks. It assesses physical health, psychological health, social relationships, and environmental context. In each of those areas, a direct score is obtained based on percentiles. The higher the score, the higher the perceived quality of life [50].

### 2.4. Statistical Analysis

The study variables were described by the treatment group and the assessment points, with frequencies and percentages for the categorical variables, and medians and percentiles of 25% and 75% were used for the continuous variables.

Following the same methodology as Mendo et al. [12], short-term effectiveness was estimated using Cliff’s delta effect size for the difference in change (post–pre) between the treatment groups (AT vs. TAU) in the continuous scales. The magnitude of the effect size can be interpreted using the thresholds provided by Romano et al. [51], meaning that |d| < 0.147 is a negligible effect size, |d| < 0.33 is a small effect size, |d| < 0.474 is a medium effect size, and anything above that is a large effect size. In addition, linear models were adjusted with the changes (post–pre) in each of the scales as a dependent variable and the study group and the pre-treatment measurements as independent variables. Scale means were reflected, when necessary, so that increasing scores over time meant improvement.

Longitudinal trends in post-treatment outcomes were analysed using multivariable mixed-effects models. Binomial distribution was assumed for dichotomized outcomes (physical activity: never/occasionally versus weekly/daily; employment situation: not active versus active), and beta distribution was assumed for continuous bounded outcomes, dividing by their ranges to obtain values between 0 and 1. The independent variables were the study group (AT vs. TAU) and months from pre-treatment (as natural splines). Sex, age, and pre-treatment measurement were included as adjusting variables. An interaction term of study group by time was included to assess the effectiveness of the intervention over the entire follow-up period.

R version 4.2.0 was used for all analyses [52]. The cliff.delta function of the effsize package [53] was used to estimate the Cliff’s delta. The mixed-model function of the GLMM adaptive package [54] was used to fit the mixed-effects models, and the results were visualized using the ggplot2 package [55].

## 3. Results

Before the start of treatment, no differences were observed between the study groups in sociodemographic characteristics (Table 1) or in clinical and psychometric outcomes (Table 2, pre-treatment assessment). However, the high proportion of patients at high risk of suicide stands out: 100% in the TAU group and 65.4% in the AT group.

It should also be noted that none of the participants in the TAU group required treatment adjustment during therapy, unlike the patients in the AT group, where 26.9% required treatment adjustment.

### 3.1. Short-Term Effectiveness

**Figure 1:**
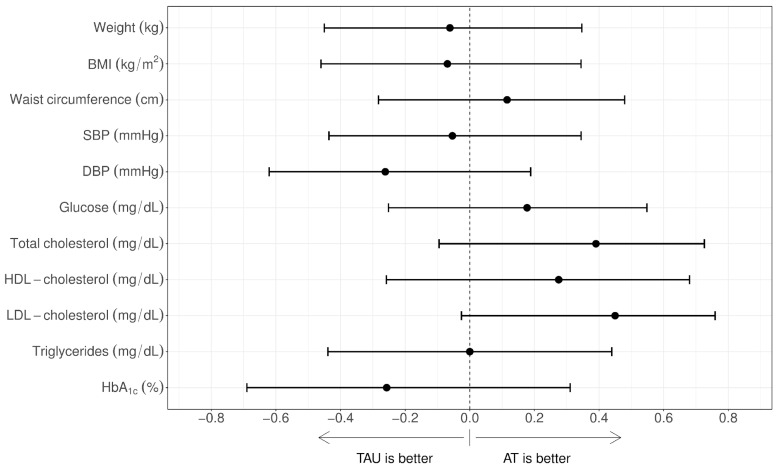
Effect sizes of the differences post–pre-treatment between groups regarding clinical variables. The values on the right indicate improvements for the AT group. AT: Adventure Therapy; TAU: treatment as usual; BMI: body mass index; SBP: systolic blood pressure; DBP: diastolic blood pressure; HDL cholesterol: high-density lipoprotein cholesterol; LDL cholesterol: low-density lipoprotein cholesterol; HbA_1c_: glycated haemoglobin.

**Figure 2:**
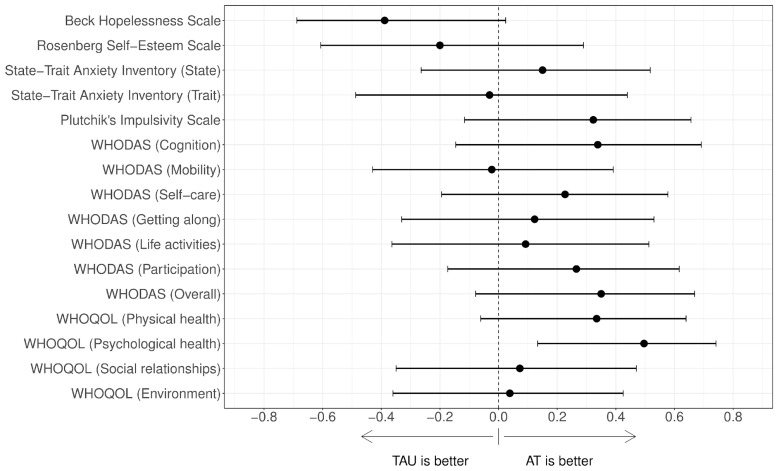
Effect sizes of the differences post–pre-treatment between groups regarding psychometric variables. The values on the right indicate improvements for the AT group. AT: Adventure Therapy, TAU: treatment as usual, WHODAS: World Health Organization Disability Assessment Schedule, WHOQOL-BREF: World Health Organization Quality of Life (short version).

Clinical and psychometric outcomes at post-treatment assessment are shown in Table 2. Effect size estimates are shown in Table 3 and Figure 1 and Figure 2. A statistically significant Cliff’s delta effect size was observed in favour of the AT group for psychological health. A medium non-significant effect size in favour of AT was observed in the WHODAS total score and physical health. A medium-large non-significant effect size in favour of AT was also observed for LDL cholesterol, which became significant after adjustment for pre-treatment measures.

### 3.2. Long-Term Effectiveness

Table 4 shows the outcomes observed at 6 months, 12 months, and 2–3 years post-therapy. The longitudinal analysis of the entire follow-up showed no significant differences between the study groups in the development of virtually any clinical or psychometric measurement, indicating that there were no long-term differences between the two therapies. However, some findings are worth noting.

No differences were found in employment over the entire follow-up period, nor in physical activity, although patients in the TAU group reported a higher frequency of moderate physical activity than those in the AT group at 2–3 years. The biochemical parameter trends showed no difference, not even in LDL cholesterol, which had shown significant short-term effects.

**Figure 3:**
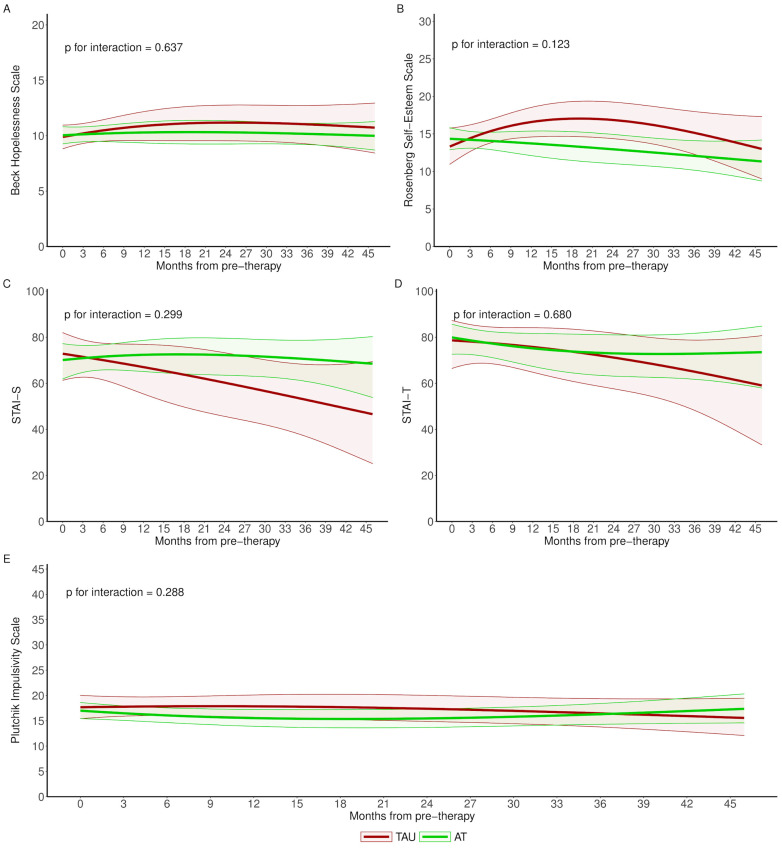
Longitudinal evolution of psychometric outcomes of an average woman for AT and TAU groups. p for interaction shows whether there are significant differences in evolution between study groups. AT: Adventure Therapy, TAU: treatment as usual, STAI: State-Trait Anxiety Inventory; Labels (**A**–**E**) are related to the different scales.

The longitudinal population means also showed very similar trends in psychometric outcomes for both study groups, with no differential impact between therapies at any time during follow-up (Figure 3).

**Figure 4:**
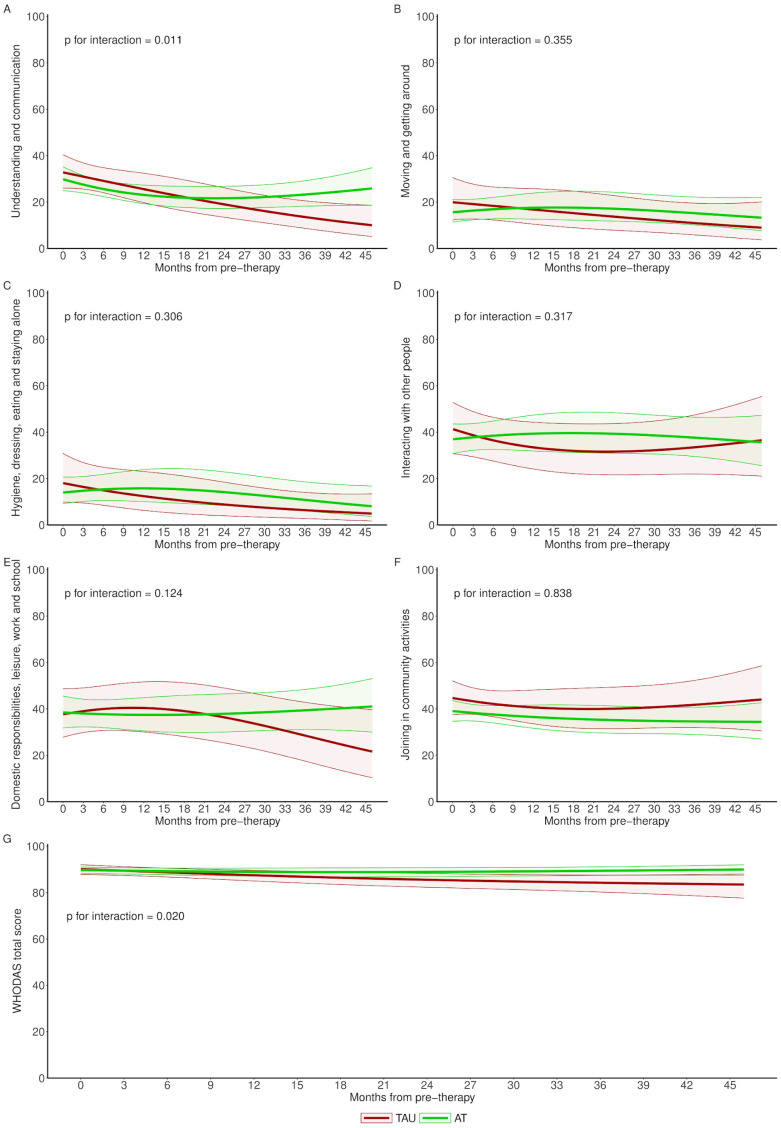
Longitudinal evolution of disability outcomes of an average woman for AT and TAU groups. p for interaction shows whether there are significant differences in evolution between study groups. AT: Adventure Therapy, TAU: treatment as usual, WHODAS: World Health Organization Disability Assessment Schedule; Labels (**A**–**G**) are related to the different scales.

It was observed that the slightly favourable differences for AT in almost all disability outcomes during the first few months of follow-up were reversed in patients with available long-term data, with even clinically non-relevant significant differences in favour of the TAU group in the cognition dimension and the overall WHODAS score (Figure 4).

**Figure 5:**
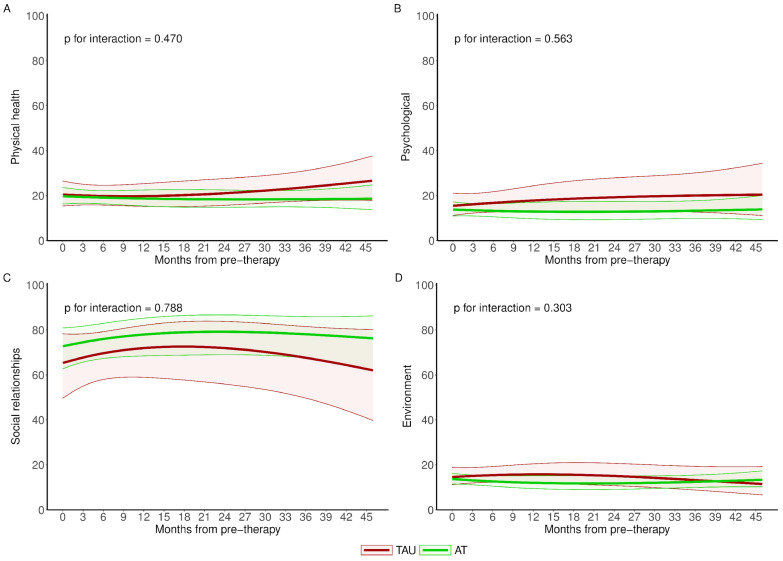
Longitudinal evolution of quality of life outcomes of an average woman for AT and TAU groups. p for interaction shows whether there are significant differences in evolution between study groups. AT: Adventure Therapy, TAU: treatment as usual, WHOQOL-BREF: World Health Organization Quality of Life (short version); Labels (**A**–**D**) are related to the different scales.

Finally, quality of life scores developed similarly in both treatment groups, without significant differences between them throughout the follow-up period (Figure 5).

## 4. Discussion

This study extended the sample of a previous study and evaluated the short- and long-term effects of AT in patients diagnosed with BPD compared with a group of patients treated with TAU in terms of their psychosocial, clinical, functional outcomes and quality of life levels. In the short term, as in the pilot study [12], many parameters showed a trend toward improvement in the AT group, especially analytical parameters and quality of life. This trend was not maintained in the long-term study, where there were no significant differences between the two treatments.

With regard to short-term sociodemographic variables, we highlighted the presence of autolytic risk in more than half of the participants throughout the study and without differences between the treatment groups. These results are consistent with the pilot study of AT in BPD patients [12]. The presence of suicide risk is one of the main symptoms of people diagnosed with BPD [3], and specific therapies are required to treat these symptoms [57].

In the longitudinal sociodemographic results, there is no change in the employment situation. Although the development time is limited, these findings are consistent with previous research concluding that people with BPD have worse psychosocial functioning, including a deterioration in work [2].

With regard to short-term clinical variables, after the therapy, the short-term study replicates the results of the study by Mendo et al. (2021) [12] and shows a trend towards healthier lifestyle habits in the AT group, together with an improvement in biological markers of physical health.

However, in the long-term study, the improvement in the results of the pre–post study was not maintained. People with BPD tend to have a greater deterioration in physical health, along with a higher prevalence of metabolic syndrome [58] and cardiovascular disease [5,6,59,60]. The use of maladaptive emotional regulation strategies (toxicity, binge eating, sexual promiscuity, poor adherence to treatment, etc.) [61] and the psychotropic drugs used to treat this disorder [62] may directly increase the likelihood of developing physical illness. In addition, dysfunction of the parasympathetic component of the autonomic nervous system (ANS) [63] and hyperstimulation of the hypothalamic–pituitary–adrenal–cortisol axis [64] have been described in these patients, increasing the likelihood of developing these diseases. Therefore, to maintain the improvements seen in the study in the short term, it could be necessary to increase the number of AT sessions. It could also be important to implement specific programmes to improve the physical health of these patients, as other studies have concluded [65,66].

With regard to the results of the psychometric variables in the pre–post results, there was a trend towards an improvement in the perception of psychological and physical quality of life and functionality in the patients in the AT group. These results are relevant because, in various studies, patients with BPD show poor quality of life [8,9] and changes in functionality [2,9] that could be improved by AT, as observed in studies using this therapeutic modality [13,67].

The trend towards improvement in the psychometric variables was lost in the long-term study. In particular, we would like to highlight the results for the functionality and quality of life scales. Given that one of the main characteristics of patients with BPD is a significant rigidity to change [3,4], it may be that the experiential learning did not have enough time to generalize during the sessions, suggesting the need for treatment with more sessions. Most studies show improvement over 24 months of treatment with 1–2 sessions per week [68].

In addition, during the clinical follow-up of these patients, the team of specialists who conducted the AT observed positive effects on intangibles, such as better coping in situations they would normally avoid [30] (improved experiential avoidance), increased tolerance to discomfort in situations of frustration and uncertainty, and also a greater use of intrapersonal or interpersonal coping strategies (asking for help, assertively expressing discomfort, etc.), which indicate an improvement in emotional regulation [29]. The psychometric tests used in this study cannot measure these improvements, so future studies would need to use scales that examine experiential avoidance and emotional regulation.

It is worth noting that the positive effects of AT could also be observed in other situations. For example, at the initial conference, patients commented on how they had incorporated the week’s learning into their daily lives, and at the debriefing at the end of each AT session, patients commented on their achievements and what they had learned. A satisfaction survey was carried out after the last session, and everyone responded positively to questions about their ability to do more things than they thought they could or reported an improvement in their ability to ask for help. These results cannot be quantified with the current study design. A qualitative methodology would allow the changes that patients verbalize and professionals observe at a clinical level to be captured in more detail. Work using this methodology has been published, evaluating young people with BPD [69,70] and dialectical behaviour therapy (DBT) [71] treatment. There are no published qualitative studies of AT.

Our research has shown that it is important to consider the therapeutic complexity of patients with this diagnosis due to their persistent autolytic risk. Additionally, the short-term improvements in physical health suggest specific actions that should be considered for future research paths, such as extending the duration of the AT programme and supplementing the treatment with psychoeducational programmes focused on physical health to improve the long-term trends observed. Additionally, it is recommended to include more specific evaluation instruments to assess the intangible effects observed during therapy and to consider using qualitative methodologies to better understand perceived changes in clinical improvement.

### Practical Issues to Consider in AT Programmes for Patients with Borderline Personality Disorder

After three editions of the AT programme, we reaffirm the practical recommendations of the article by Mendo et al. (2021) [12] regarding the need for therapists with experience in the knowledge and management of the disorder, the existence of a prior therapeutic alliance with the patient, the need for support therapists (at least two) to contain crisis situations during sessions, and the importance of a device with quick and direct access to deal with crisis interventions that may occur during the programme. In this sense, we have already mentioned that more than a quarter of the AT patients required pharmacological adjustment, especially at the beginning of the programme.

The growing experience of professionals in this therapeutic modality in successive editions has allowed for the planning of activities with a higher perceived risk (and greater need for emotional management). For example, due to their similarity to the emotional difficulties that patients must manage throughout their lives (metaphor), a kayaking activity and a via *ferrata* were carried out, activities that, once started, have to be continued until the end. Both posed great difficulties for the patients, who had to manage avoidance and emotional regulation when frustration, discomfort, or impulsivity arose. The strategies used to overcome the difficulties in these activities were very useful in redirecting situations of emotional difficulty in the patients’ subsequent evolution.

## 5. Limitations

This study has limitations due to the mental health pathology of our sample, the limited sample size, the assessment instruments used, and the time period during which the information was collected, which was during the COVID-19 pandemic [37].

One of the main limitations of the study is the loss of patients in the long-term study. Several factors may have influenced this, including the personality traits inherent to the disorder [3] (instability, impulsiveness, difficulty in therapeutic engagement, etc.), and the pandemic period of COVID-19 [37], which made it impossible to collect 18-month data from patients. This fact was taken into account in our analyses.

Caution is necessary when interpreting the results of this study for several reasons. The results obtained in the short-term study, as in the study by Mendo et al. (2021) [12], could be related to changes in habits and to the individual variability of biochemical parameters in blood tests. In addition, we need to be cautious in interpreting these trends and take into account the size of the sample. The loss of improvement in long-term studies makes us consider the need to evaluate a longer duration of AT in order to consolidate the changes obtained in the short term while also making us consider the need to introduce psychoeducation programmes related to physical health in this group of patients.

We would like to stress the importance of re-evaluating the instruments used to assess changes brought about by AT. The long-term psychometric results contrast with the clinical evolution of the patients who completed the AT programmes. Better emotional management and less experiential avoidance were observed, concepts that cannot be measured with the scales used in this study. In order to assess the clinical improvement observed in these patients, it would be necessary to use experiential avoidance assessment scales such as the AAQ-II (Acceptance and Action Questionnaire) [72,73], as well as emotional management scales such as the DERS (Difficulties in Emotion Regulation Scale) [74,75]. In addition, there are no specific instruments to measure the changes that occur in AT [15].

Finally, in line with what has been mentioned above, this limitation could also be overcome by adopting a qualitative methodology in the study of these patients.

## 6. Conclusions

In conclusion, it is important to note that our study does not allow us to affirm that AT produces better results than TAU, since the positive effects observed immediately after the therapy seemed to be attenuated in the long term. Therefore, the effectiveness of the long-term psychotherapy did not show differences between TA and TAU therapies for the treatment of patients with BPD, that is, no conclusive evidence was found to suggest that TA is superior to TAU. However, the effects of intangibles observed during therapy by professionals and patients themselves were not reflected in the measures collected.

For this reason, we believe it is necessary to increase the duration of the AT programme, complement the treatment with a specific physical health programme, assess the results with more specific instruments, and/or move towards a qualitative methodology to measure the perceived changes in clinical improvement in these patients. New studies will be needed to assess the results of the proposed changes.

## Figures and Tables

**Table 1:** Baseline characteristics of the study population per treatment group.

	Adventure Therapy (AT) N = 26	Treatment as Usual (TAU) N = 10
Female	17 (65.4)	6 (60)
Age (years)	39.5 (31.8, 45.0)	39.0 (29.2, 51.2)
Marital status		
*Single*	13 (50)	8 (80)
*Married*	8 (30.8)	2 (20)
*Separated/divorced*	3 (11.5)	0 (0)
*Others*	2 (7.69)	0 (0)
Cohabitation situation		
*Live alone*	6 (23.1)	2 (20)
*Lives with family*	14 (53.8)	6 (60)
*Cohabiting*	6 (23.1)	1 (10)
*Others*	0 (0)	1 (10)
Educational level		
*Primary*	2 (7.69)	2 (20)
*Secondary*	12 (46.2)	5 (50)
*University*	12 (46.2)	3 (30)
Profession		
*No profession*	3 (11.5)	3 (30)
*Student*	4 (15.4)	2 (20)
*Non-qualified labour*	5 (19.2)	1 (10)
*Qualified work*	14 (53.8)	4 (40)
Employment situation		
*Active*	6 (23.1)	2 (20)
*Sick leave*	9 (34.6)	2 (20)
*Unemployed*	6 (23.1)	4 (40)
*Pensioner*	5 (19.2)	2 (20)
Secondary diagnosis		
*Bipolar affective disorder*	1 (4)	1 (11.1)
*Unipolar affective disorder*	11 (44)	6 (66.7)
*Schizoaffective disorder*	0 (0)	1 (11.1)
*Anxiety Disorder*	4 (16)	1 (11.1)
*Adaptive disorder*	3 (12)	0 (0)
*Substance use disorder*	1 (4)	0 (0)
*Eating Disorder*	3 (12)	0 (0)
*Others*	2 (8)	0 (0)
Tobacco	8 (30.8)	4 (40)
Other toxic		
*None*	20 (76.9)	9 (90)
*Alcohol*	4 (15.4)	1 (10)
*Cannabis*	2 (7.69)	0 (0)

Categorical variables described with frequencies and percentages by study group. For age, median and percentiles of 25% and 75% are shown.

**Table 2:** Descriptive table of the pre- and post-treatment assessments per treatment group.

	Pre-Treatment Assessment		Post-Treatment Assessment	
	Adventure Therapy (AT) N = 26	Treatment as Usual (TAU) N = 10	Adventure Therapy (AT) N = 26	Treatment as Usual (TAU) N = 10
Weight (kg)	78.6 (66.1, 91.2)	77.4 (74.2, 91.6)	79.0 (67.7, 92.2)	80.0 (66.8, 92.5)
BMI (kg/m^2^)	25.9 (23.2, 30.7)	29.5 (25.4, 31.3)	25.7 (23.6, 32.8)	28.2 (24.5, 33.1)
Waist circumference (cm)	95.8 (86.2, 107)	98.0 (94.5, 105)	93.0 (86.5, 105)	99.5 (91.2, 106)
SBP (mmHg)	124 (110, 140)	128 (122, 134)	126 (115, 132)	120 (114, 135)
DBP (mmHg)	83.5 (70.2, 90.0)	88.0 (78.2, 92.0)	82.5 (77.5, 87.8)	82.5 (77.8, 86.0)
Glucose (mg/dL) (74–100 ^a^)	89.5 (86.2, 94.0)	94.0 (83.8, 95.8)	90.0 (82.0, 98.5)	93.5 (85.5, 102)
Total cholesterol (mg/dL) (150–220 ^a^)	208 (177, 229)	196 (180, 221)	204 (183, 236)	216 (186, 236)
HDL cholesterol (mg/dL) (45–84 ^a^)	52.0 (44.5, 57.0)	47.0 (42.0, 53.0)	53.0 (45.0, 57.0)	44.5 (42.5, 48.8)
LDL cholesterol (mg/dL) (65–160 ^a^)	130 (104, 154)	120 (109, 139)	116 (110, 152)	144 (109, 160)
Triglycerides (mg/dL) (50–200 ^a^)	106 (87.8, 158)	160 (112, 166)	119 (100, 161)	132 (104, 200)
HbA_1c_ (%) (4.6–5.8 ^a^)	5.20 (5.10, 5.43)	5.20 (5.00, 5.20)	5.40 (5.20, 5.65)	5.20 (5.10, 5.60)
Physical activity				
*Never*	8 (30.8)	2 (20)	5 (19.2)	2 (20)
*Occasionally*	8 (30.8)	3 (30)	3 (11.5)	4 (40)
*Weekly*	7 (26.9)	3 (30)	11 (42.3)	2 (20)
*Daily*	3 (11.5)	2 (20)	7 (26.9)	2 (20)
Beck Hopelessness Scale	10.0 (8.00, 11.8)	11.0 (9.50, 12.0)	10.5 (8.25, 12.8)	11.0 (7.50, 12.0)
*Low risk of suicide (0–8)*	9 (34.6)	0 (0)	7 (26.9)	3 (30)
*High risk of suicide (9–20)*	17 (65.4)	10 (100)	19 (73.1)	7 (70)
Rosenberg Self-Esteem Scale	14.5 (11.2, 19.0)	12.5 (9.25, 13.8)	14.0 (12.0, 19.8)	15.0 (12.2, 17.0)
*Low self-esteem (0–14)*	13 (50)	8 (80)	14 (53.8)	5 (50)
*Normal esteem (15–30)*	13 (50)	2 (20)	12 (46.2)	5 (50)
STAI				
*STAI State*	76.0 (55.0, 89.0)	78.5 (28.5, 88.0)	75.0 (51.2, 83.8)	77.5 (45.0, 88.0)
*STAI Trait*	88.0 (65.0, 97.0)	82.5 (38.8, 95.0)	85.0 (66.2, 95.8)	70.0 (62.5, 78.8)
Plutchik Impulsivity Scale	19.5 (12.0, 24.8)	16.0 (14.2, 18.5)	16.5 (12.2, 26.8)	17.5 (13.8, 23.8)
*Low impulsivity (0–20)*	13 (50)	9 (90)	15 (57.7)	6 (60)
*High impulsivity (21–45)*	13 (50)	1 (10)	11 (42.3)	4 (40)
WHODAS				
*Understanding and communication*	33.3 (20.8, 54.2)	33.3 (22.9, 43.7)	31.2 (20.8, 40.6)	40.8 (26.0, 47.9)
*Mobility*	17.5 (5.00, 40.0)	25.0 (11.2, 25.0)	22.5 (6.25, 33.8)	22.5 (11.2, 28.8)
*Personal care*	12.5 (1.19, 25.0)	12.5 (6.25, 23.4)	6.25 (0.00, 23.4)	12.5 (6.25, 18.2)
*Relationships*	42.5 (15.0, 53.8)	33.5 (20.0, 43.8)	37.5 (20.0, 58.8)	37.5 (20.0, 52.3)
*Daily life activities*	37.5 (18.8, 46.9)	34.4 (24.2, 46.9)	40.6 (15.6, 50.0)	32.8 (18.8, 40.6)
*Participation in society*	46.9 (32.0, 55.4)	39.1 (28.9, 49.2)	40.6 (22.7, 46.9)	42.2 (28.1, 43.8)
*Total score*	88.3 (82.7, 90.4)	87.1 (85.8, 90.4)	87.1 (82.7, 90.4)	88.1 (83.5, 90.4)
WHOQOL-BREF				
*Physical Health*	13.0 (13.0, 25.0)	19.0 (14.5, 19.0)	19.0 (13.0, 25.0)	13.0 (13.0, 19.0)
*Psychological*	13.0 (6.00, 28.0)	19.0 (13.0, 23.5)	19.0 (6.00, 31.0)	19.0 (13.0, 19.0)
*Social relationships*	56.5 (31.0, 79.5)	69.0 (50.0, 69.0)	69.0 (44.0, 75.0)	69.0 (51.5, 75.0)
*Environment*	19.0 (13.0, 19.0)	13.0 (13.0, 19.0)	19.0 (13.0, 25.0)	16.0 (13.0, 19.0)

Categorical variables described with frequencies and percentages for the study group. For continuous variables, median and percentiles of 25% and 75% are shown. BMI: body mass index, SBP: systolic blood pressure, DBP: diastolic blood pressure, HbA_1c_: glycated haemoglobin, STAI: State-Trait Anxiety Inventory, WHODAS: World Health Organization Disability Assessment Schedule, WHOQOL-BREF: World Health Organization Quality of Life (abbreviated version). Normal values: BMI (18.5–24.9 kg/m^2^) [56]; waist circumference (F < 88 cm; M < 102 cm) [56]; SBP (<130 mmHg) [56]; DBP (<85 mmHg) [56]; glucose (74–100 mg/dL) ^a^; total cholesterol (150–220 mg/dL) ^a^; HDL cholesterol (45–84 mg/dL) ^a^; LDL cholesterol (65–160 mg/dL) ^a^; triglycerides (50–200 mg/dL) ^a^; HbA_1c_ (%) (4.6–5.8%) ^a^. ^(a)^ Normal values according to laboratory.

**Table 3:** Effect size and adjusted mean difference between groups (AT vs. TAU) of the changes post–pre-treatment ^a^.

	Cliff’s Delta (95% CI)		Mean Difference ^b^ (95% CI)	
Weight (kg)	−0.06	(−0.45, 0.35)	−0.95	(−4.02, 2.11)
IMC (kg/m^2^)	−0.07	(−0.46, 0.34)	−0.42	(−1.53, 0.69)
Waist circumference	0.12	(−0.28, 0.48)	0.45	(−3.48, 4.38)
SBP (mmHg)	−0.05	(−0.44, 0.34)	−0.59	(−9.34, 8.17)
DBP (mmHg)	−0.26	(−0.62, 0.19)	−3.61	(−11.8, 4.57)
Glucose (mg/dL)	0.18	(−0.25, 0.55)	0.93	(−10.6, 12.4)
Total cholesterol (mg/dL)	0.39	(−0.09, 0.73)	13.96	(−1.99, 29.9)
HDL cholesterol (mg/dL)	0.28	(−0.26, 0.68)	3.55	(−1.62, 8.72)
LDL cholesterol (mg/dL)	0.45	(−0.03, 0.76)	15.11	(0.75, 29.5)
Triglycerides (mg/dL)	0.00	(−0.44, 0.44)	1.21	(−26.5, 28.9)
HbA_1c_ (%)	−0.26	(−0.69, 0.31)	−0.10	(−0.93, 0.72)
Beck Hopelessness Scale	−0.39	(−0.69, 0.02)	−1.27	(−3.18, 0.65)
Rosenberg Self-Esteem Scale	−0.20	(−0.61, 0.29)	−1.41	(−4.79, 1.98)
STAI State	0.15	(−0.26, 0.52)	4.97	(−9.61, 19.6)
STAI Trait	−0.03	(−0.49, 0.44)	1.07	(−12.1, 14.2)
Plutchik Impulsivity Scale	0.32	(−0.12, 0.66)	2.28	(−0.93, 5.50)
WHODAS				
*Understanding and communication*	0.34	(−0.15, 0.69)	5.66	(−2.64, 14.0)
*Mobility*	−0.02	(−0.43, 0.39)	0.68	(−7.49, 8.84)
*Personal care*	0.23	(−0.19, 0.58)	1.67	(−11.8, 15.2)
*Relations*	0.12	(−0.33, 0.53)	2.17	(−9.74, 14.1)
*Daily life activities*	0.09	(−0.36, 0.51)	−0.44	(−10.7, 9.83)
*Participation in society*	0.27	(−0.17, 0.62)	6.29	(−1.70, 14.3)
*Total score*	0.35	(−0.08, 0.67)	1.81	(−1.81, 5.42)
WHOQOL-BREF				
*Physical Health*	0.33	(−0.06, 0.64)	5.73	(−0.19, 11.7)
*Psychological*	0.50	(0.13, 0.74)	5.48	(0.43, 10.5)
*Social relationships*	0.07	(−0.35, 0.47)	1.32	(−14.7, 17.3)
*Environment*	0.04	(−0.36, 0.42)	−2.99	(−9.94, 3.96)

^a^ Scale means are reflected when necessary; increased scores over time meant an improvement. Positive values mean that AT is better than TAU, both for effect size and mean difference. ^b^ Mean difference between group changes (post–pre) adjusted by the pre-treatment measure. AT: Adventure Therapy, TAU: treatment as usual, CI: confidence interval, BMI: body mass index, SBP: systolic blood pressure, DBP: diastolic blood pressure, HbA_1c_: glycated haemoglobin, STAI: State-Trait Anxiety Inventory, WHODAS: World Health Organization Disability Assessment Schedule, WHOQOL-BREF: World Health Organization Quality of Life (short version). Significant effects in bold. The magnitude of the effect size can be interpreted using the thresholds provided in Romano et al. (2006) [51]: |d| < 0.147 is a negligible effect size, |d| < 0.33 is a small effect size, |d|< 0.474 is a medium effect size, and |d| ≥ 0.474 is a large effect size.

**Table 4:** Descriptive table of the assessments at 6 months, 12 months, and 2–3 years post-treatment per treatment group.

	6-Month Assessment		12-Month Assessment		2–3 Years Assessment	
	Adventure Therapy (AT) N = 10	Treatment as Usual (TAU) N = 9	Adventure Therapy (AT) N = 9	Treatment as Usual (TAU) N = 7	Adventure Therapy (AT) N = 18	Treatment as Usual (TAU) N = 4
Weight (kg)	85.7 (71.7, 93.4)	81.8 (62.2, 89.5)	82.9 (68.2, 91.5)	73.5 (62.5, 81.7)	81.5 (71.2, 94.5)	99.0 (89.4, 105)
BMI (kg/m^2^)	28.7 (25.0, 32.8)	27.0 (24.3, 31.2)	28.0 (23.9, 30.3)	25.4 (23.7, 28.6)	27.9 (25.0, 30.9)	33.4 (30.0, 36.5)
Waist circumference (cm)	100 (89.2, 112)	96.0 (85.0, 98.5)	96.0 (86.0, 104)	94.0 (83.2, 96.8)	99.5 (91.8, 106)	109 (98.8, 119)
SBP (mmHg)	132 (128, 138)	119 (112, 126)	126 (120, 130)	110 (106, 114)	134 (121, 143)	142 (138, 146)
DBP (mmHg)	84.0 (80.2, 88.8)	73.0 (70.0, 80.0)	83.0 (76.0, 84.0)	71.0 (65.0, 77.5)	87.0 (82.2, 93.8)	93.0 (87.2, 98.8)
Glucose (mg/dL)	88.0 (79.0, 89.0)	87.0 (84.8, 93.8)	82.0 (81.0, 85.0)	82.0 (78.2, 86.5)	95.0 (87.2, 106)	97.0 (90.8, 108)
Total cholesterol (mg/dL)	209 (201, 211)	202 (186, 229)	217 (200, 236)	201 (189, 234)	205 (188, 217)	199 (196, 212)
HDL cholesterol (mg/dL)	62.0 (45.0, 64.0)	49.5 (43.0, 54.2)	53.5 (48.5, 62.0)	51.0 (44.8, 55.0)	54.0 (45.5, 58.0)	45.5 (43.5, 47.2)
LDL cholesterol (mg/dL)	138 (126, 160)	134 (120, 155)	150 (135, 177)	127 (117, 153)	121 (106, 140)	130 (127, 141)
Triglycerides (mg/dL)	96.0 (81.0, 147)	125 (74.8, 142)	95.0 (72.0, 138)	151 (116, 158)	114 (77.0, 139)	138 (121, 151)
HbA_1c_ (%) (4.6–5.8 ^a^)	5.40 (5.20, 5.40)	5.20 (5.07, 5.38)	5.30 (5.25, 5.45)	5.10 (5.00, 5.40)	5.40 (5.15, 5.53)	5.40 (5.00, 5.60)
Physical activity						
*Never*	1 (10)	3 (33.3)	1 (11.1)	2 (28.6)	3 (16.7)	0 (0)
*Occasionally*	0 (0)	2 (22.2)	1 (11.1)	1 (14.3)	7 (38.9)	0 (0)
*Weekly*	6 (60)	3 (33.3)	5 (55.6)	2 (28.6)	4 (22.2)	3 (75)
*Daily*	3 (30)	1 (11.1)	2 (22.2)	2 (28.6)	4 (22.2)	1 (25)
Beck Hopelessness Scale	10.0 (8.25, 11.8)	12.0 (10.0, 13.0)	11.0 (9.00, 13.0)	13.0 (10.5, 13.0)	11.0 (8.00, 12.0)	12.0 (10.2, 13.0)
*Low risk of suicide (0–8)*	3 (30)	1 (11.1)	1 (11.1)	0 (0)	6 (33.3)	1 (25)
*High risk of suicide (9–20)*	7 (70)	8 (88.9)	8 (88.9)	7 (100)	12 (66.7)	3 (75)
Rosenberg Self-Esteem Scale	12.5 (11.0, 14.8)	15.0 (13.0, 15.0)	15.0 (13.0, 16.0)	15.0 (13.5, 18.0)	13.0 (11.0, 14.0)	13.0 (11.8, 14.5)
*Low self-esteem (0–14)*	7 (70)	4 (44.4)	4 (44.4)	3 (42.9)	15 (83.3)	3 (75)
*Normal esteem (15–30)*	3 (30)	5 (55.6)	5 (55.6)	4 (57.1)	3 (16.7)	1 (25)
STAI						
*STAI State*	70.0 (37.5, 85.0)	55.0 (40.0, 80.0)	65.0 (45.0, 83.0)	75.0 (57.5, 82.0)	78.5 (43.8, 85.0)	32.5 (12.2, 58.8)
*STAI Trait*	72.5 (36.2, 95.0)	77.0 (65.0, 85.0)	65.0 (45.0, 89.0)	60.0 (56.5, 90.0)	80.0 (46.2, 95.0)	52.5 (20.0, 81.2)
Plutchik Impulsivity Scale	11.5 (10.0, 20.8)	16.0 (13.0, 19.0)	14.0 (11.0, 23.0)	20.0 (14.0, 21.5)	17.0 (11.0, 22.0)	16.5 (13.0, 19.0)
*Low impulsivity (0–20)*	7 (70)	7 (77.8)	6 (66.7)	3 (42.9)	11 (61.1)	4 (100)
*High impulsivity (21–45)*	3 (30)	2 (22.2)	3 (33.3)	4 (57.1)	7 (38.9)	0 (0)
WHODAS						
*Understanding and communication*	10.4 (8.33, 29.2)	29.2 (16.7, 37.5)	20.8 (12.5, 41.2)	33.3 (20.8, 43.8)	31.2 (16.7, 39.6)	10.4 (3.13, 21.9)
*Mobility*	15.0 (2.50, 36.2)	20.0 (10.0, 40.0)	10.0 (5.00, 15.0)	20.0 (12.5, 35.0)	15.0 (5.00, 37.5)	7.50 (3.75, 16.2)
*Personal care*	15.6 (1.56, 35.9)	6.25 (0.00, 18.8)	6.25 (0.00, 12.5)	6.25 (6.25, 18.8)	15.6 (0.00, 25.0)	0.00 (0.00, 3.12)
*Relationships*	25.0 (16.2, 30.0)	20.0 (15.0, 35.0)	20.0 (15.0, 30.0)	30.0 (22.5, 45.0)	35.0 (20.0, 53.8)	32.5 (17.5, 45.0)
*Daily life activities*	32.8 (20.3, 49.2)	37.5 (18.8, 40.6)	18.8 (18.8, 43.7)	37.5 (28.1, 42.2)	42.2 (28.1, 55.5)	17.2 (10.9, 22.7)
*Participation in society*	26.6 (14.1, 46.9)	34.4 (31.2, 40.6)	25.0 (21.9, 40.6)	34.4 (32.8, 48.4)	35.9 (19.5, 48.4)	35.9 (26.6, 44.5)
*Total score*	82.1 (73.9, 88.3)	85.8 (78.4, 90.4)	78.4 (72.3, 82.7)	85.8 (85.8, 89.4)	85.8 (79.5, 93.6)	77.5 (72.3, 83.5)
WHOQOL-BREF						
*Physical Health*	19.0 (14.5, 28.0)	13.0 (6.00, 19.0)	25.0 (13.0, 31.0)	13.0 (13.0, 28.0)	19.0 (7.75, 23.5)	19.0 (11.2, 29.8)
*Psychological*	22.0 (13.0, 31.0)	19.0 (13.0, 31.0)	19.0 (19.0, 25.0)	13.0 (13.0, 25.0)	19.0 (13.0, 29.5)	25.0 (22.0, 29.8)
*Social relationships*	69.0 (54.8, 93.8)	69.0 (50.0, 81.0)	69.0 (50.0, 81.0)	69.0 (56.5, 97.0)	72.0 (44.0, 79.5)	56.5 (33.0, 76.8)
*Environment*	22.0 (19.0, 25.0)	19.0 (13.0, 19.0)	19.0 (19.0, 25.0)	19.0 (9.50, 22.0)	19.0 (14.5, 25.0)	19.0 (11.2, 25.0)

Categorical variables described with frequencies and percentages for the study group. For continuous variables, median and percentiles of 25% and 75% are shown. BMI: body mass index, SBP: systolic blood pressure, DBP: diastolic blood pressure, HbA_1c_: glycated haemoglobin, STAI: State-Trait Anxiety Inventory, WHODAS: World Health Organization Disability Assessment Schedule, WHOQOL-BREF: World Health Organization Quality of Life (abbreviated version). Normal values: BMI (18.5–24.9 kg/m^2^) [56]; waist circumference (F < 88 cm; M < 102 cm) [56]; SBP (<130 mmHg) [56]; DBP (<85 mmHg) [56]; glucose (74–100 mg/dL) ^a^; total cholesterol (150–220 mg/dL) ^a^; HDL cholesterol (45–84 mg/dL) ^a^; LDL cholesterol (65–160 mg/dL) ^a^; triglycerides (50–200 mg/dL) ^a^; HbA_1c_ (%) (4.6–5.8%) ^a^. ^(a)^ Normal values according to laboratory.

## Data Availability

The datasets presented in this article are not readily available because they are part of an ongoing study. Requests to access the datasets should be directed to the corresponding author.
